# Loss of the Actin Remodeler Eps8 Causes Intestinal Defects and Improved Metabolic Status in Mice

**DOI:** 10.1371/journal.pone.0009468

**Published:** 2010-03-02

**Authors:** Arianna Tocchetti, Charlotte Blanche Ekalle Soppo, Fabio Zani, Fabrizio Bianchi, Maria Cristina Gagliani, Benedetta Pozzi, Jan Rozman, Ralf Elvert, Nicole Ehrhardt, Birgit Rathkolb, Corinna Moerth, Marion Horsch, Helmut Fuchs, Valérie Gailus-Durner, Johannes Beckers, Martin Klingenspor, Eckhard Wolf, Martin Hrabé de Angelis, Eugenio Scanziani, Carlo Tacchetti, Giorgio Scita, Pier Paolo Di Fiore, Nina Offenhäuser

**Affiliations:** 1 Fondazione Instituto FIRC di Oncologia Molecolare, Milan, Italy; 2 Dipartimento di Medicina, Chirurgia ed Odontoiatria, Universita' degli Studi di Milano, Milan, Italy; 3 Department of Experimental Medicine, University of Genoa, Genoa, Italy; 4 German Mouse Clinic, Helmholtz Zentrum München, Munich/Neuherberg, Germany; 5 Molecular Nutritional Medicine, Technische Universität München, Freising-Weihenstephan, Germany; 6 Institute of Molecular Animal Breeding and Biotechnology, Ludwig Maximilians Universität München, Munich, Germany; 7 Lehrstuhl für Experimentelle Genetik, Technische Universität München, Freising-Weihenstephan, Germany; 8 Facoltà di Medicina Veterinaria, Università degli Studi di Milano, Milan, Italy; 9 Istituto Europeo di Oncologia, Milan, Italy; University Medical Center Groningen, The Netherlands

## Abstract

**Background:**

In a variety of organisms, including mammals, caloric restriction improves metabolic status and lowers the incidence of chronic-degenerative diseases, ultimately leading to increased lifespan.

**Methodology/Principal Findings:**

Here we show that knockout mice for Eps8, a regulator of actin dynamics, display reduced body weight, partial resistance to age- or diet-induced obesity, and overall improved metabolic status. Alteration in the liver gene expression profile, in behavior and metabolism point to a calorie restriction-like phenotype in Eps8 knockout mice. Additionally, and consistent with a calorie restricted metabolism, Eps8 knockout mice show increased lifespan. The metabolic alterations in Eps8 knockout mice correlated with a significant reduction in intestinal fat absorption presumably caused by a 25% reduction in intestinal microvilli length.

**Conclusions/Significance:**

Our findings implicate actin dynamics as a novel variable in the determination of longevity. Additionally, our observations suggest that subtle differences in energy balance can, over time, significantly affect bodyweight and metabolic status in mice.

## Introduction

Obesity is associated with an increased risk of numerous co-morbidities, such as type 2 diabetes, hypertension and cardiovascular diseases, collectively referred to as metabolic syndrome, and cancer [1], [2], [3]. The mechanisms involved are being clarified, following the seminal discovery that adipose tissue, far from being a passive reservoir for the accumulation of lipids, is an endocrine organ that produces dozens of factors that regulate several aspects of organism homeostasis [1], [2], [3]. Consistently, mounting evidence links the production of pro-inflammatory factors, by excess adipose tissue, to the development of a systemic chronic inflammatory state that contributes to the development of obesity-associated morbidities [1], [2], [3].

Studies in mice carrying fat-specific disruption of the insulin receptor gene (FIRKO mice) demonstrated how the insulin signaling pathway plays a major role in the homeostasis of adipose tissue, while its subversion leads to pathological conditions. FIRKO mice display 50% reduction in adipose tissue, despite normal food intake [4], and are protected from age-related and hyperphagia-induced obesity, and obesity-related glucose intolerance [4]. These metabolic changes correlate with extended longevity [5], thus corroborating the idea that reduced fat mass, even in the presence of normal or increased food intake, can reduce obesity-associated metabolic alterations and extend lifespan. The notion that insulin signaling in adipose tissue is critical in the regulation of lifespan received further support by findings that sirtuin 1 (SIRT1), encoded by the mammalian orthologue of SIR2 - a yeast life-extending gene -, inhibits adipogenesis by repressing PPAR-γ, an insulin-dependent master regulator of fat cell development [6].

It has been known for many years that caloric restriction (CR), with adequate nutrition, improves cardiometabolic health, prevents tumorigenesis, and increases life span in experimental animals [7]. One relevant issue is whether CR exerts its beneficial effects *per se* or through the reduction of adipose tissue mass. Studies in FIRKO mice [4], [5] support the latter possibility, while studies in genetic models of CR have not yet addressed the issue. Here, we report one such a model, eps8-null (Eps8KO) mice [8], [9], which unexpectedly links actin dynamics to individual variations in bodyweight, metabolic status and longevity.

Eps8 is a multimodular protein involved in actin remodeling [9], [10], [11], [12], [13] through several activities, including regulation of Rac, a pivotal GTPase in the control of actin dynamics [9], [14], and direct interaction with actin. Through this latter property, Eps8 exerts both actin barbed end capping and actin bundling activities [11], [12]. We systematically analyzed Eps8KO mice to unmask possible defects pointing to phenotypes related to human disease. This analysis revealed that Eps8KO mice weigh less than WT mice and are partially resistant to aging and diet-induced obesity. Moreover, Eps8KO mice display a CR phenotype, accompanied by increased insulin sensitivity and an improved metabolic status, which are likely responsible for the increased lifespan observed in Eps8KO mice. This negative energy balance in Eps8KO mice is not caused by decreased food intake or increased energy expenditure, instead it correlates with decreased intestinal absorption and reduced intestinal microvilli length. Thus, the actin remodeler Eps8 is required for proper microvilli morphogenesis and loss of Eps8 in mice leads to altered intestinal function, improved metabolism and increased lifespan.

## Results

### Eps8KO Mice Are Predisposed to Leanness

**Figure 1:**
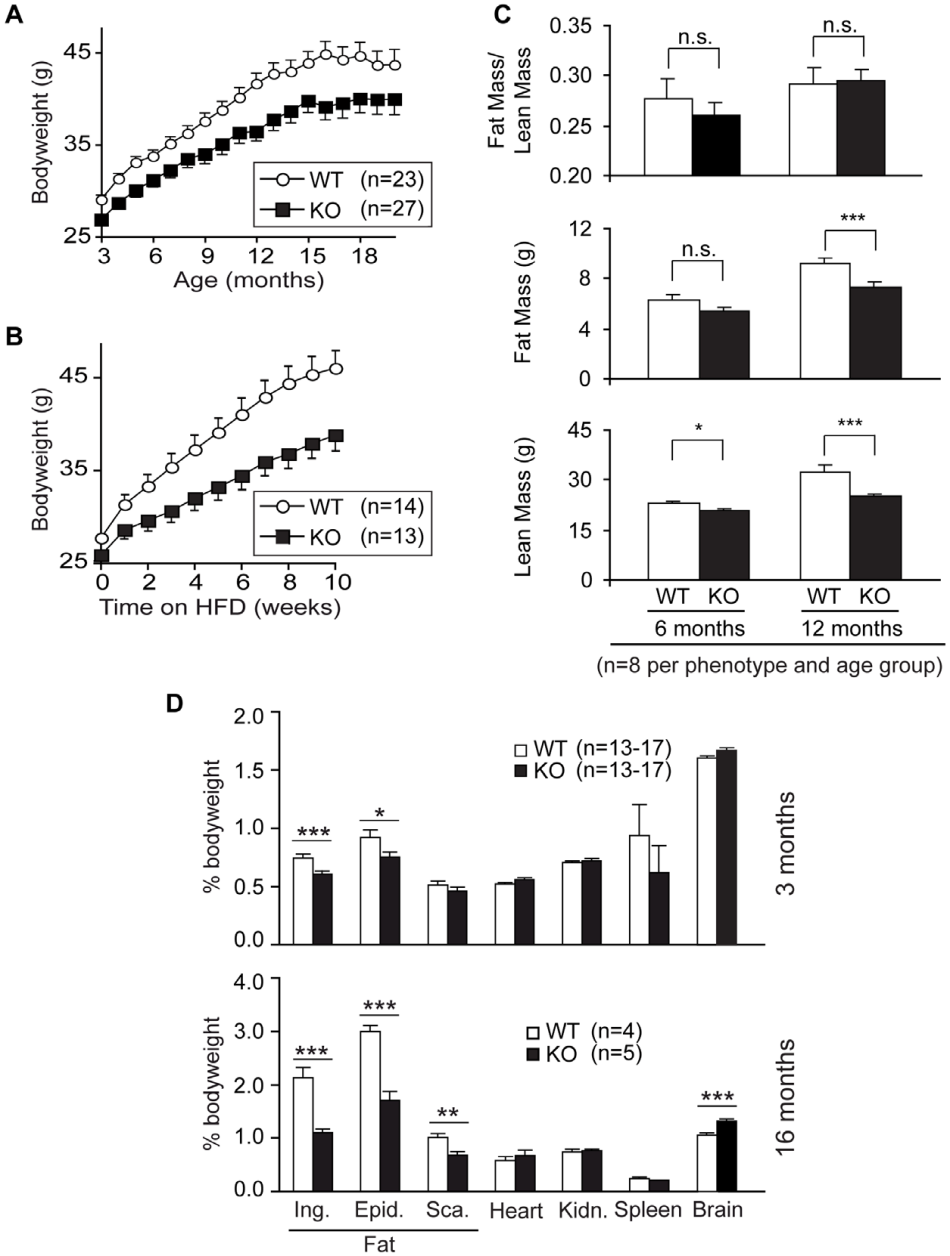
Reduced bodyweight in Eps8KO mice. **A–B.** Age- (A) and high fat diet-induced (B) obesity in Eps8KO and wild-type mice (KO and WT, respectively, in this and all subsequent figures). HFD, high fat diet. **C.** Soxhlet analysis. **D.** Weight of individual organs. Ing., inguinal white fat; Epid., epididymal white fat; Sca., scapular brown fat; Kidn., kidney. Values are expressed as percent bodyweight. In all panels (and in all subsequent figures): n, number of mice tested per condition (in D, 13–17 mice were used, depending on the analyzed organ). In all panels (and in all subsequent figures), values are expressed as means ± SEM. Two-tailed t-test was used to assess statistical significance: *, *P*<0.05; **, *P*<0.01; ***, *P*<0.005; n.s., not significant (in this and in all subsequent figures).

Eps8KO mice displayed a significant reduction in bodyweight, when compared to wild-type (WT) mice, something that became much more evident during aging (Fig. 1A) or high-fat diet-induced obesity (Fig. 1B). To gain insights into the nature of these phenomena, we used Soxhlet analysis to measure whole body fat content and lean body mass in Eps8KO mice. Reduced bodyweight was due to a reduction in both the fat and the lean mass in young and, more pronouncedly, in old Eps8KO mice (Fig. 1C). When the weight of individual organs was measured as a fraction of total body weight, however, we observed that for the majority of inner organs there was no significant difference between Eps8KO and WT mice. Instead, a significant reduction in inguinal, epididymal and scapular fat, relative to total body weight, was observed in Eps8KO mice (Fig. 1D). Importantly, total brain weight was unaffected in Eps8KO mice, which resulted in an increase of its fractional contribution to body weight, in older mice (Fig. 1D).

We concluded that Eps8KO mice show a lean phenotype. In addition, results from the detailed measurements of organ weight are compatible with the possibility that these mice are functionally calorie-restricted. Mice subjected to alimentary CR, in fact, show an absolute reduction in both lean and fat mass, and a relatively higher proportional loss of fat mass, while the brain is the sole organ that does not show a reduction in weight [15], [16]. The possibility that Eps8KO mice are calorie-restricted will be further analyzed, and discussed, in a following section.

### Improved Metabolic Status in Eps8KO Mice

**Figure 2:**
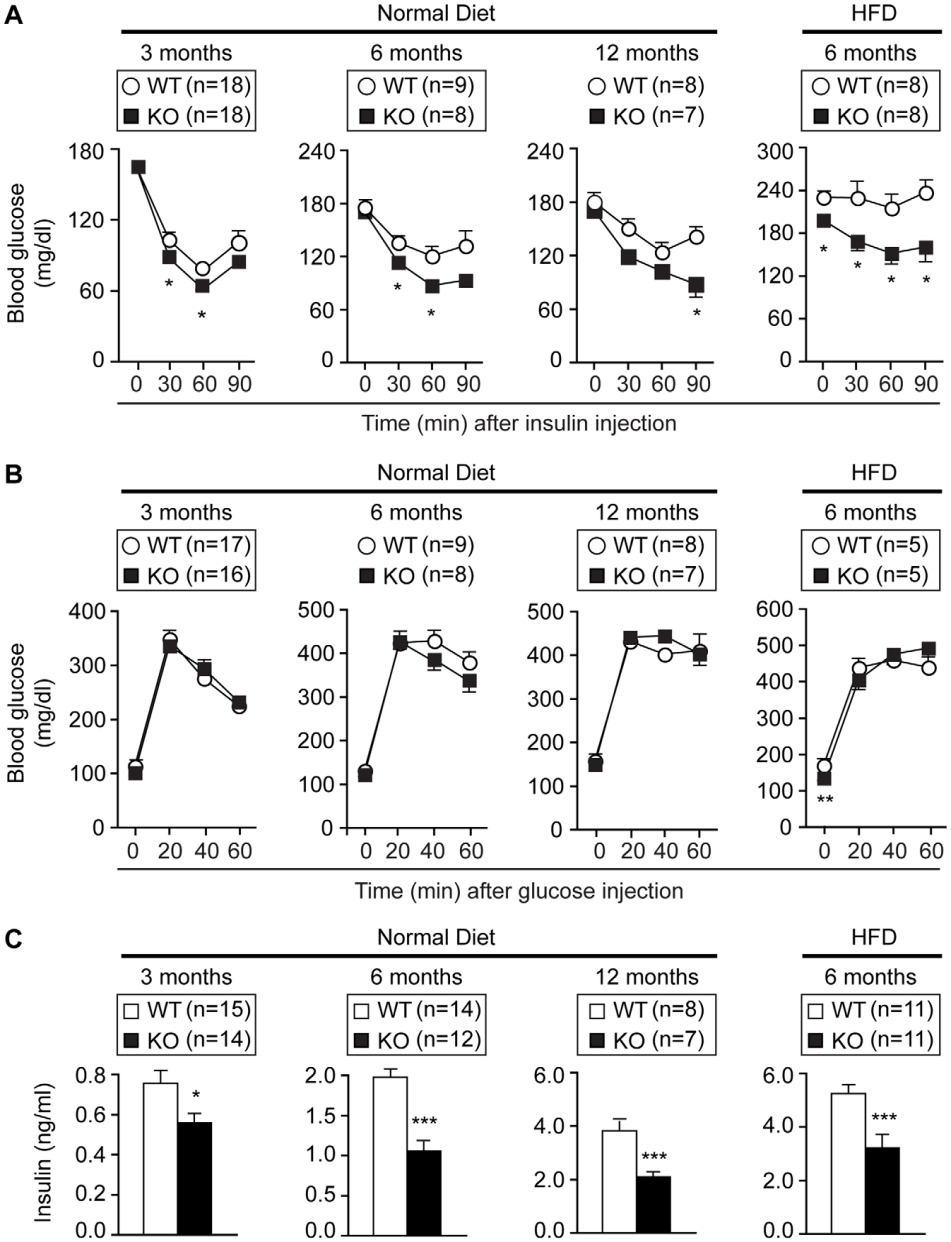
Increased insulin sensitivity in Eps8KO mice. **A.** Insulin tolerance, measured as blood glucose after insulin injection. **B.** Glucose tolerance, measured as blood glucose after glucose injection. **C.** Plasma insulin levels after overnight fast. HFD, high fat diet.

Increased bodyweight correlates inversely with insulin sensitivity and overall metabolic status, which in turn affects the risk of type 2 diabetes and of cardiovascular diseases [17], [18]. Accordingly, Eps8KO mice displayed increased insulin sensitivity both at a young and an older age and after high fat diet (Fig. 2A); however, they displayed no increased glucose tolerance (Fig. 2B). Insulin levels were lower in Eps8KO mice at all ages and for all dietary regimes (Fig. 2C). Thus, in Eps8KO mice, reduced bodyweight both during aging- and diet- induced obesity correlates with improved insulin sensitivity.

We next compared metabolic parameters of Eps8KO and WT mice (Table S1). While only insulin and leptin levels were reduced in Eps8KO mice fed a normal diet (8% reduction in bodyweight compared to WT mice), after a high fat diet (20% reduction in bodyweight), Eps8KO mice additionally displayed significantly reduced glucose, triglyceride and cholesterol values. A similar improvement in blood parameters was observed in Eps8KO mice after an overnight fast (Table S1). We concluded that Eps8KO mice display an overall improved metabolic status.

### Eps8KO Mice Display Reduced Fat Absorption

**Figure 3:**
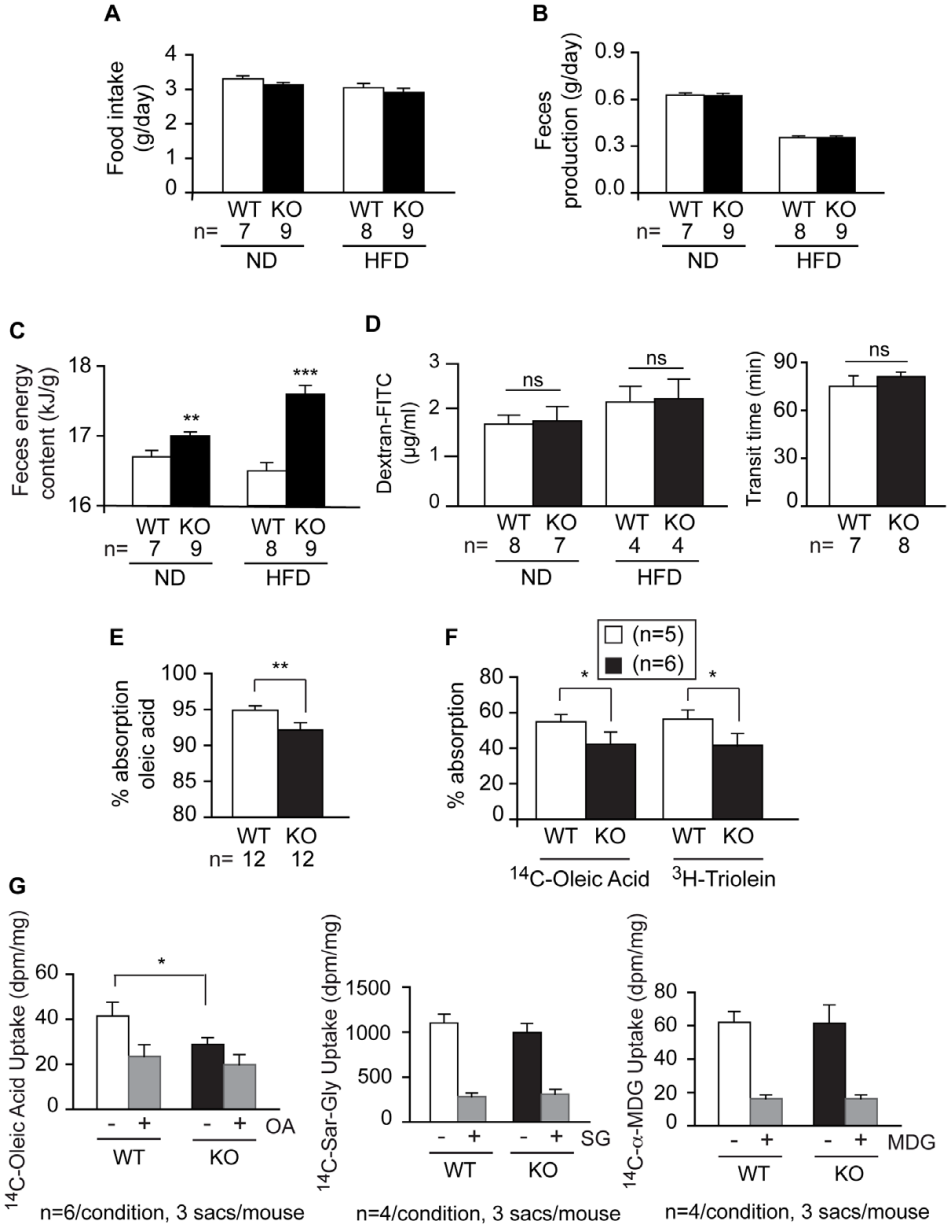
Reduced intestinal fat absorption in Eps8KO mice. **A.** Food intake. **B.** Feces production. **C.** Feces energy content. **D.** Left, intestinal permeability, determined fluorometrically in the plasma 4 hr after an oral gavage of Dextran-FITC (4 kD). Right, *in vivo* intestinal transit, determined as the time (transit time) between oral gavage of Carmin Red and the appearance of red feces. **E.** Absorption of ^14^C-oleic acid determined by the fecal dual isotope method. **F.** Intestinal fat absorption determined by the plasma dual isotope method. Please note that while differences are readily detectable between genotypes, no difference in absorption is observable between the two substrates, within genotypes. **This** indicates that the breakdown of triglycerides at the intestinal level is not impaired. **G.** Intestinal uptake of a fatty acid (left), peptidic (middle), and sugar (right) substrate determined by the everted intestinal sac model. Grey bars depict background values after competition with excess cold substrate: OA, 40 mM cold oleic acid; SG, 20 mM cold sarcosyl-glycine, MDG, 100 mM cold methyl-glucopyranoside. In each experiment 3 sacs/mouse were prepared from the number of mice/experimental condition indicated in the figure. In all panels: ND, normal diet; HFD, high fat diet.

One possible cause of reduced bodyweight is reduced food intake/assimilation. We did not detect significant differences in food intake in Eps8KO mice, either when fed on normal chow or on a high fat diet (Fig. 3A). Similarly, we did not detect alterations in the amount of feces produced by Eps8KO mice (Fig. 3B). Instead, determining the calorific value of the feces using a combustion calorimeter, we found that the feces of Eps8KO mice displayed a higher energy content than those of normal mice, both on a normal and on a high fat diet (Fig. 3C). This increase in fecal energy content was not due to altered intestinal permeability or intestinal transit time (Fig. 3D).

Collectively, the above data suggest that Eps8KO mice absorb less. To investigate this possibility directly, we initially measured fat absorption, since increased fecal calorie content was more pronounced when Eps8KO mice were fed a high fat diet. Using the fecal dual isotope method in which the non-absorbable sitostanol serves as an internal normalizer, we observed a significant reduction in oleic acid absorption in Eps8KO mice (Fig. 3E). Next we measured intestinal absorption using the plasma dual isotope method in which plasma lipases were inhibited to allow accumulation of the absorbed lipids. Oleic acid and triolein (a triglyceride analog) absorption was equally reduced, indicating that the action of intestinal lipases is not impaired in Eps8KO mice (Fig. 3F). To address whether the absorption deficit was specific for fat or whether absorption of other nutrients was also impaired in Eps8KO mice, we used the everted sac model. Uptake of ^14^C-MDG, ^14^C-Gly-Sar was similar in WT and Eps8KO mice, while ^14^C-oleic acid uptake was reduced in intestinal sacs from Eps8KO mice (Fig. 3G). Permeability to ^3^H-mannitol was negligible throughout the experiment (Fig. S1). Thus, while sugar and peptide absorption is normal, fat absorption is specifically impaired in Eps8KO mice.

### Energy Expenditure of Eps8KO Mice

The above data show that reduced food assimilation, in particular fat, is associated with the lean phenotype of Eps8KO mice. However, increased energy expenditures might also contribute to this phenotype.

**Figure 4:**
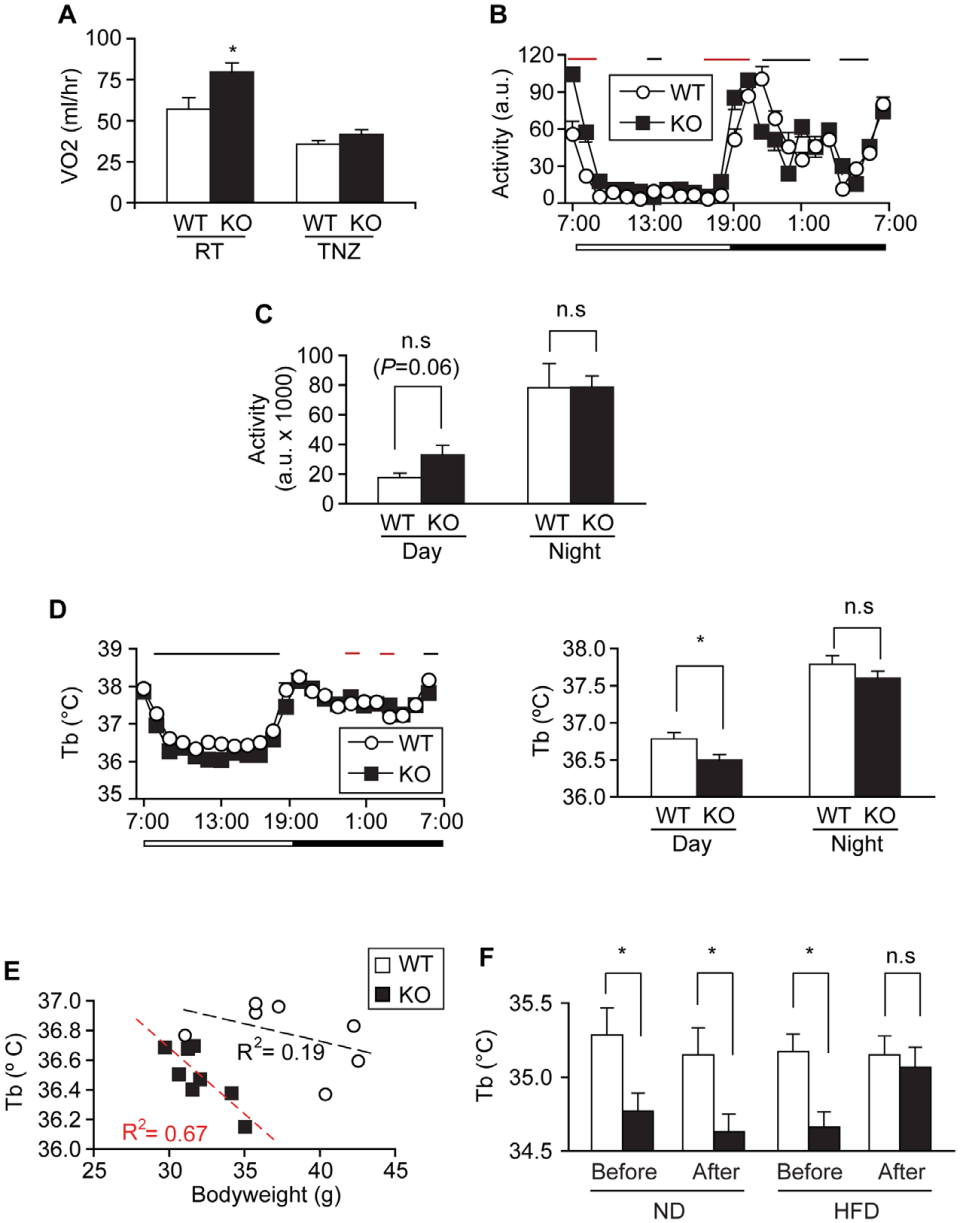
Energy expenditure of Eps8 KO mice. **A.** Oxygen consumption (VO_2_) determined at ambient temperature (RT), and at 30°C (TNZ, thermal neutral zone). **B–C.** Locomotor activity determined on a 24 hr cycle (B) or represented as the average of day and night time (C). In panel B (and in the following panel D), time points, that differed significantly between WT and Eps8KO mice are indicated by horizontal lines. Red and black lines indicate values that were higher or lower, respectively, in Eps8KO vs. WT mice. **D.** Core body temperature, determined telemetrically in WT and Eps8KO mice, and presented as a 24 hr cycle (top) or as the average of day and night time (bottom). **E.** Scatter plot of the correlation between core body temperature and bodyweight. **F.** Core body temperature before and after 8 weeks on normal chow (ND) or on high fat diet (HFD). In all graphs n = 7 for WT and 8 for KO.

Daily energy expenditure, determined as oxygen consumption at room temperature, was increased in Eps8KO mice (Fig. 4A). Next, we measured the metabolic rate at 30°C (TNZ, thermal neutral zone), where energy expenditure for thermal regulation is minimal. Under these conditions, we did not detect any significant difference between WT and KO mice (Figure 4A). We also measured locomotor activity. On a 24 hr schedule, activity was not significantly increased in Eps8KO mice (Fig. 4B), although we noticed a trend towards increased activity during the light phase (*P* = 0.06), but not during the dark phase, in Eps8KO mice (Fig. 4C).

This initial set of results indicated that there is increased energy expenditure in Eps8KO mice (Fig. 4A). However, this could not be immediately ascribed to increased locomotor activity (Fig. 4B–C) or to increased metabolic rate when the need for thermogenesis was minimized at the TNZ (Fig. 4A). One possibility, to explain the increased energy expenditure is that Eps8KO mice invest more than WT in thermoregulation, possibly due to reduced insulation. To gain more insights into this issue, we measured core body temperature (Tb). In Eps8KO mice, there was reduced Tb during the light but not during the dark phase (Fig. 4D). In addition, we found that, in Eps8KO mice, core body temperature inversely correlated with body weight, while in WT mice no such correlation was observed (Fig. 4E).

These results suggest that, in Eps8KO mice, energy resources are limited and individual mice allocate available energy resources preferentially either into fat storage or into thermogenesis. To test this hypothesis directly, we fed mice a high fat diet and measured core body temperature at the beginning and at the end of the experiment. As expected, under conditions of increased caloric intake, core body temperature in Eps8KO mice rose to levels of WT mice (Fig. 4F), while it remained unvaried in mice fed a normal diet. These results further support the hypothesis that limited energy resources are at the base of the reduced core body temperature in Eps8KO mice. Interestingly, reduced core body temperature is a phenotype also displayed by calorie-restricted mice [19], [20], an observation that further supports the possibility that Eps8KO mice might be functionally calorie-restricted.

### Eps8KO Mice Are Calorie-Restricted

To corroborate our hypothesis, we employed several approaches. Calorie-restricted mice show a characteristic gene expression profile, reflecting alterations in metabolic pathways due to reduced availability of nutrients [21], [22], [23]. We analyzed the gene expression profiles in livers from Eps8KO and WT mice. Eps8 is not expressed in liver [24], thus differences in gene expression should reflect metabolic changes rather than the direct effect of the lack of Eps8. We found 20 genes differentially expressed in between WT and Eps8KO livers (cut-off ± 1.6 fold, *P*<0.05, Table S2). We validated, by quantitative PCR, nine genes (four upregulated and five downregulated in the Eps8KO/WT comparison), and found 100% concordance (Table S2).

**Figure 5:**
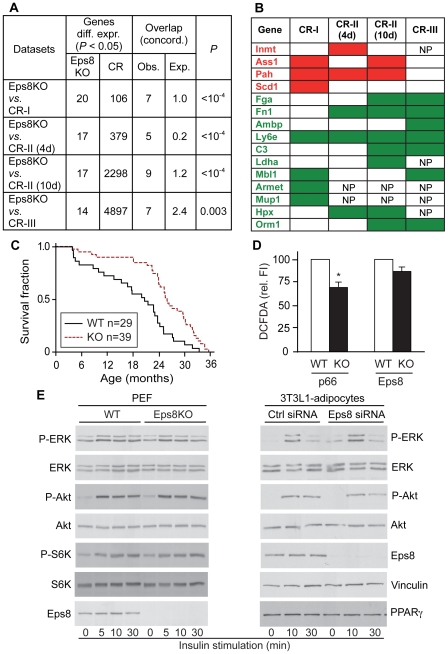
Eps8KO mice display a CR-like phenotype. **A.** The 20 genes differentially expressed in the livers of Eps8KO mice vs. WT (See Table S2) were compared to published studies that analyzed differential gene expression in the livers of CR mice compared to controls [CR-I, [21]; CR-II, [22]; CR-III, [23]]. In the study by Pohjanvirta *et al*., two conditions of starvation (4 days and 10 days) were used, and both are reported in the comparison. In the study by Bauer *et al*., several conditions were used and we report the longest one (48 hs) in the comparison. “Genes differ. expr. (*P*<0.05)”, number of significantly (*P*<0.05) differentially expressed genes in this study (Eps8KO) and in the other studies (CR) (the number of Eps8KO genes vary because not all genes that we analyzed were present on the chips used in the other studies). “Overlap (concord.)”, genes overlapping between the indicated datasets and concordantly regulated (upregulated or downregulated); the observed (Obs.) overlapping genes are reported in comparison to the expected (Exp.) overlap in a random distribution. “*P*”, p-value of the observed overlap vs. the expected one (Pearsons's chi-squared test). Additional details are in the Materials and Methods section. **B.** Detailed analysis of the overlap between genes from the Eps8KO list (column “Gene”; in red, upregulated genes, in green, downregulated genes) with CR lists from other studies (CR-I, CR-II and CR-III as in A). Only concordant overlaps are shown and are indicated by a red box if the gene was upregulated, or by a green box if the gene was downregulated. Genes not present in a specific array experiment are indicated as NP **C.** Kaplan-Meyer survival curves for Eps8KO mice and WT littermates. **D.** ROS production as measured by DCFDA fluorescence in primary embryo fibroblasts (PEF) from Eps8KO and p66KO mice and WT littermates. p66KO fibroblasts [48] were used as positive controls. **E.** Insulin signaling. Left, PEF from Eps8KO or WT mice were treated with Insulin 10 µg/ml for the indicated times, followed by immunoblot (IB) as shown. Right, 3T3-L1 adipocytes were subjected to Eps8 silencing (eps8 siRNA) or to mock-silencing (ctrl siRNA) followed by Insulin treatment and IB as in left.

We then compared this expression profile with a number of liver expression profiles published for the CR phenotype of mice [21], [22], [23]. As shown in Fig. 5A, there was a very significant overlap between the genes differentially expressed in livers of Eps8KO vs. WT mice and those differentially expressed in livers of CR vs. control mice. In addition, 15 of the 20 genes of our profile were concordantly and significantly differentially expressed in at least one of four published CR datasets (Fig. 5B, see also Table S3).

It should be noted that in our expression profiling analysis the number of significantly differentially expressed genes was lower than in the published studies that we used for our comparisons. One obvious possibility is that differences in the profiling platforms employed in the various studies account for the differences. It should also be noted that Eps8KO mice represent a “chronic” condition of calorie restriction, in which adaptive changes might have occurred. In addition, Eps8KO are only moderately and selectively calorie-restricted (the absorption defect is fat-specific). The other studies reported conditions of acute (from 24 h to 8 weeks) and massive calorie restriction (starvation), in which metabolic and gene expression changes are more likely to be of a vaster magnitude. Regardless, the metabolic changes occurring in the livers of Eps8KO mice closely resemble those occurring under condition of CR.

If Eps8KO mice were indeed functionally calorie-restricted they should display increased longevity [7]. Indeed Eps8KO lived significantly longer than WT mice (Fig. 5C). The median survival of Eps8KO mice was increased by 26% (*P* = 0.005), mean survival by 37% (*P* = 0.00071) and maximum survival by 9% (*P* = 0.012). Lifespan was significantly extended both in male and female Eps8KO mice (Fig. S2A–B). Increased lifespan in genetically modified mice has been linked to either a reduction in free radical production or alterations in the insulin signaling pathway [25]. Moreover, Eps8 has recently been suggested to control ROS production directly [26]. However, we did not detect altered ROS production in primary fibroblasts (Fig. 5D) or in macrophages (Fig. S2C) derived from Eps8KO mice. Similarly, we did not detect alterations in insulin signaling in fibroblasts or adipocytes devoid of Eps8 (Fig. 5E). We concluded that the longevity phenotype of Eps8KO mice is not due to direct alterations of ROS production or insulin signaling, and it can therefore most likely be ascribed to the functional CR obtained in these animals.

### A Microvillar Morphogenetic Defect in Eps8KO Mice

**Figure 6:**
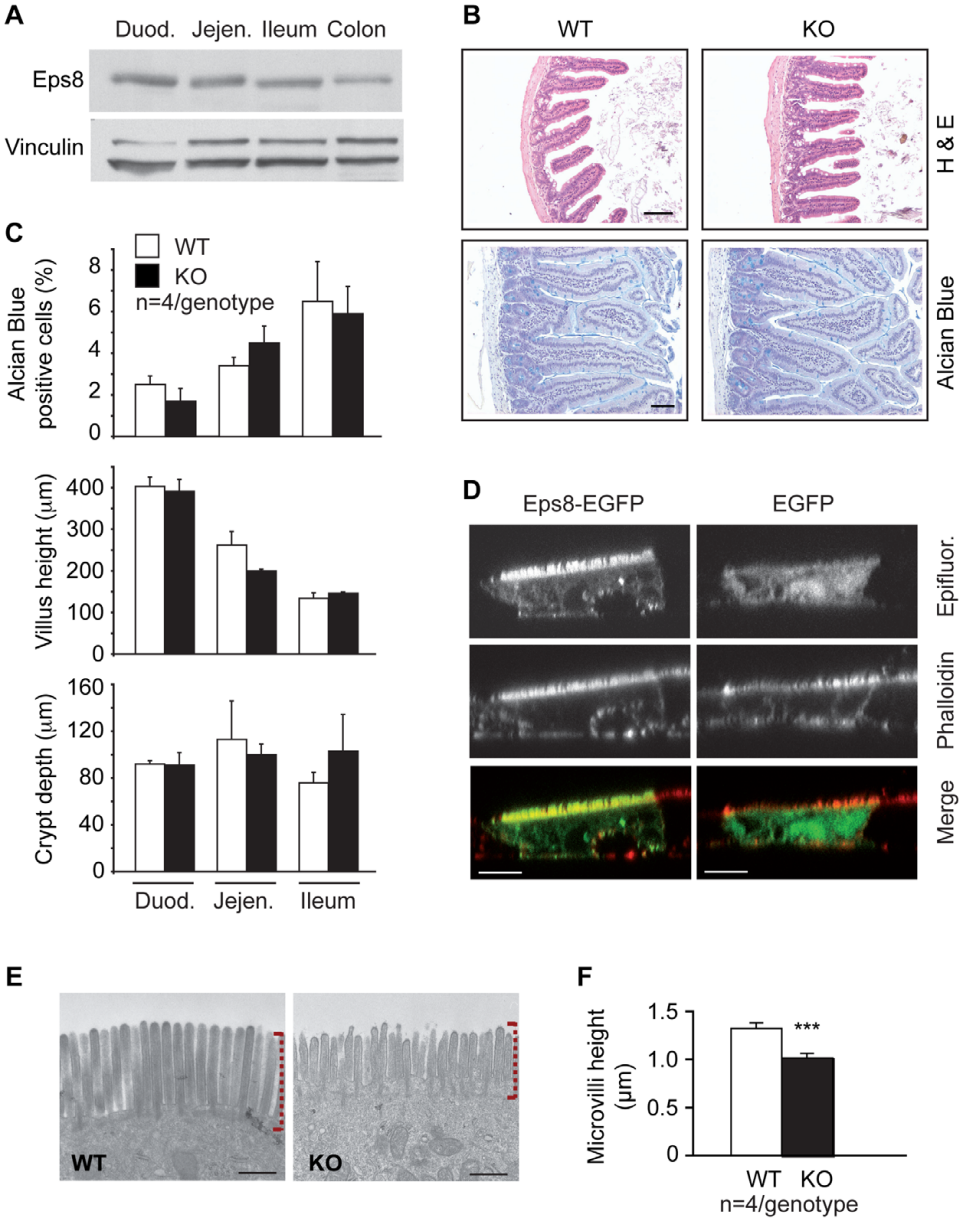
Eps8 is required for correct microvillar morphogenesis in the mouse. **A.** IB analysis of Eps8 in the intestine. Duod., duodenum, Jejen., jejunum, Ile. **B.** Small intestines (jejunum) from WT or KO mice were stained with hematoxylin-eosin (H&E, top) to assess general morphology, or Alcian blue (bottom) to visualize goblet cells. Bar, 100 µm. **C.** Morphometric analysis of Alcian blue positive cells (top), villus height (middle) and crypt depth (bottom) in the indicated intestinal segments. **D.** Confocal images of Caco-2 cells, transfected with Eps8 (EGFP-Eps8, right) or control EGFP (EGFP, left). Cells were allowed to differentiate for 10 days on collagen-coated coverslips, and then stained for F-actin using rhodaminated phalloidin. The merged images (merge. Bottom panels) show the colocalization (yellow) of EGFP-Eps8 or EGFP (green) with F-actin (red). Bar, 10 µm. **E.** Transmission electron microscope analysis of the intestinal brush border demonstrates shortened and irregular shaped microvilli (dashed red brackets) in the duodenal tracts of KO compared to WT mice. Bar, 0.5 µm. **F.** Morphometric analysis of duodenal microvilli length.

We searched for the possible causes of functional CR in Eps8KO mice. Since these mice absorb less fat, we directed our attention to possible intestinal alterations. In the mouse, Eps8 is expressed in the bowel, both in the small intestine and, to a lesser extent, in the colon (Fig. 6A). Histological examination of the small intestine did not reveal gross differences between Eps8KO and WT mice (Fig. 6B), and a morphometric analysis showed no differences in villus length, crypt height, or number of alcian-blue positive goblet cells (Fig. 6C). Similarly, no evident alterations were detectable in the large intestine of Eps8KO mice (data not shown).

These results suggest that more subtle changes might be responsible for the phenotype of Eps8KO mice. We analyzed the subcellular localization of Eps8 in intestinal cells. We found that EGFP-Eps8, but not EGFP alone, colocalizes with F-actin to the apical membrane of differentiated intestinal Caco-2 cells, suggesting that Eps8 is localized to intestinal microvilli (Fig. 6D). Microvillar localization of Eps8 was confirmed by biochemical fractionation of the intestinal brush border membrane (Fig. S3). Thus, we analyzed the morphology of intestinal enterocytes of Eps8KO mice at the ultrastructural level. Microvilli in Eps8KO enterocytes appeared disorganized and were significantly shorter than their WT counterparts (Figure 6E–F). This was not accompanied by a general disorganization of the actin cytoskeleton as assessed by phalloidin staining for F-actin (data not shown). Since microvilli serve to augment the absorptive surface of the intestine, their reduction in Eps8KO mice is compatible with the absorption defect and with the calorie restriction phenotype that we observed in these animals.

## Discussion

In this study, we show that the genetic removal in mice of a regulator of actin dynamics, Eps8, leads to a complex phenotype characterized by leanness, improved metabolic status and increased lifespan. All these phenotypes most likely rest on the fact that Eps8KO mice are functionally calorie-restricted.

Mechanistically, our data are compatible with a model in which the lack of Eps8 causes an alteration in microvillar morphogenesis and hence in the absorptive function of the intestinal brush border. The altered absorptive function of the intestine in turn, leads to a limited but constant reduction in energy intake, which is not compensated by increased food intake. The first result of the reduced energy intake is a reduction in bodyweight with consequent leanness. The reduction in weight is, in part, due to loss of adipose tissue. The concomitant reduction in insulation leads to a greater caloric demand for thermoregulation, which, when combined with the reduced energy intake, causes a CR-like state in the mice over time. As a consequence, insulin sensitivity is increased, glucose levels are lowered, and core body temperature decreases. These phenotypes are characteristic of an improved metabolic status that is most likely responsible for the increased lifespan detected in Eps8KO mice. While this scenario needs further experimental confirmation, first and foremost the reproduction of the phenotype in an intestine-specific KO strain, our results unexpectedly link a well-characterized regulator of actin dynamics to individual variations in bodyweight, metabolic status and longevity, and mark this process as a potential co-determinant in the pathogenesis of obesity and of its co-morbidities.

### Eps8 Is Essential for Proper Microvillar Morphogenesis

Our findings identify the process of microvillar morphogenesis as the most likely initial step in the determination of the lean phenotype of Eps8KO mice. How does Eps8 control this process? Microvilli are highly dynamic structures undergoing constant remodeling through actin treadmilling. Consistently, even when microvilli have reached their final length and become bundled by cross-linkers, actin monomers are continuously added at the barbed end of the actin filaments, facing the microvilli tips, and dissociate from the pointed end [27]. By controlling this process, actin binding and bundling proteins are crucial regulators of microvillar length and architectural organization, respectively.

Eps8 is a multimodular actin remodeling protein, exerting both actin barbed end capping and F-actin cross-linking and bundling activity. The switch between these activities is regulated through interaction with its binding partners: association with Abi-1 activates Eps8's capping, association with Irsp53 its bundling activity [11], [12]. A further degree of regulation is exerted by phosphorylation regulating both Eps8's association with F-actin as well as its capping activity [28]. Thus, Eps8 represents an actin remodeler that can be dynamically regulated to modulate its function. In C.elegans, the bundling activity of Eps8 is essential for microvilli formation, whereas the capping activity is dispensable [29]. Whether the same holds true for mammals needs to be demonstrated. The fact that the capping activity of mouse Eps8 is 20-fold higher than the one of worm Eps8 [10] might suggest that for mammalian microvilli morphogenesis not only the bundling but also the capping activity is needed. It is worth pointing out that in addition to Eps8 two other Eps8 gene family members, that display similar biochemical activities of Eps8, are expressed in the intestine [24], suggesting a further and unexplored level of complexity in the regulation of microvilli growth. Single and combined knockout mice for the various Eps8 family members will be needed to dissect the specific and redundant functions of the Eps8 family members in microvilli morphogenesis.

How do other actin binding and bundling proteins fit into this picture? *In vitro* work in cell lines had suggested an important role for Villin and Espin, two F-actin bundling proteins, enriched in the intestinal brush border, in microvillus morphogenesis [30], [31], [32]. Instead, neither knockout mice show any microvillar alterations, suggesting that, *in vivo* the loss of these two proteins is compensated by other F-actin bundlers. Instead, loss of Formin (Plastin 1), an F-actin bundler, which additionally, through its interaction with Keratin19, links the microvillar actin rootlets to the terminal web, leads to shortened microvilli and reduced transmembrane resistance in mice [33]. Surprisingly, Formin knockout mice do not show any metabolic alterations. Since in these mice reduced microvilli length is accompanied by increased permeability, it is tempting to speculate that the two alterations balance each other, leading net to normal nutrient uptake.

### Determination of the Calorie-Restricted Phenotype of Eps8KO Mice

Based on our data, we propose that the initiating event in the generation of the complex phenotype of Eps8KO mice is the reduction of the intestinal surface area available for nutrient absorption. However, we detected a specific fat absorption defect in Ep8KO mice, while sugar and peptides were equally well absorbed in comparison to WT mice. One possible explanation for the selectiveness of the defect might reside in the fact that sugars and peptides are transported across the apical surface of enterocytes via specific carriers. Such active transport mechanisms might compensate for the reduction of the transport area, either by increasing their rate or their concentration (although we did not measure these variables directly). Conversely, fatty acids enter via free diffusion, and thus a reduction in surface area would directly impinge on their influx rate.

A number of issues require further discussion. First, why do Eps8 mice not compensate the reduced absorption by eating more? Food intake is centrally regulated [34] and Eps8 is expressed in the brain [8], raising the possibility that alterations in the CNS are at the basis of the phenotype. Although, we did not directly investigate this possibility, it is worth noting that we have previously shown that neuronal Eps8 controls at least one type of behavioral response, i.e. that to ethanol [8]. Other, not mutually exclusive, possibilities must also be contemplated. For instance, gut signals - both hormonal and nutrients - feed back to the brain to regulate food intake [35]; it is possible that the lack of Eps8 could impinge on the regulation of gut-derived signaling to control food intake. Finally, Eps8KO mice might not feel very hungry. The reduction in intestinal absorption is fat specific meaning that normal circulating levels of glucose and amino acids are still available to signal satiety.

It should also be noted that, despite the fact that Eps8KO mice do not eat more than WT littermates in absolute terms, they do so, when food intake is normalized to bodyweight. This possibly reflects the fact that they react, at least partially, to lower levels of leptin and insulin. The fact that the differences that we observe are rather small raises another interesting point: is the absorption defect sufficient to explain the CR phenotype? The effect of CR on lifespan is observed at 20–40% reduction in food intake [36], a condition not likely mimicked by the absorption defect of Eps8KO mice. Thus, other factors possibly contribute to the negative energy balance. From our data, the prime suspect is heat loss, consequent to reduced thermoinsulation due to diminished fat mass, as also supported by the finding that Eps8KO mice invest more than WT littermates for thermoregulation. Reduced absorption and increased heat loss would lead to a slow, but progressive, negative energy balance responsible for the functional CR of Eps8KO mice.

Finally, and compatibly with their CR phenotype, Eps8KO mice display improved metabolic status, which is the most likely cause of their increased lifespan. Indeed, we could not evidence any alterations in insulin signaling or in ROS production in primary cells derived from Eps8KO mice. This finding argues that Eps8 does not play a direct role in the classical pathways implicated in the regulation of lifespan [25], and that the longevity phenotype is likely a consequence of the functional CR of these mice. In further support of this possibility, we could evidence alterations in the gene expression profile of livers from Eps8KO mice that were similar to those detectable under conditions of CR [21], [22], [23]. The absence of Eps8 expression in the liver [24] definitely rules out a direct impact of Eps8 on biochemical pathways potentially leading to CR and increased lifespan, and further supports the notion that these phenotypes can be ascribed to the improved metabolic status of the KO mice.

### Implications

Our findings raise the possibility that actin dynamics might play a previously unsuspected role in the co-determination of obesity and of its associated morbidities. Whether this correlation reflects a real occurrence in humans is presently a matter of speculation; however, based on our results, an investigation of polymorphisms in the *Eps8* gene, which might point to possible hypomorphic alleles, seems warranted. Such an analysis should be extended to other Eps8-family members, since in principle hypomorphic alleles of these genes might have a similar impact to that of *Eps8* on microvillar morphogenesis and on the ensuing phenotypes.

Our data suggest that small changes in the daily energy balance are sufficient to gradually improve the metabolic state and prolong lifespan. This observation is important as it is generally thought that severe caloric restriction of 20–40% is necessary to achieve such effects [36]. Our findings might open perspectives for much more feasible therapeutic strategies than the rigorous dieting regimens that few people are able to follow.

## Materials and Methods

### Animal Housing, Diet and Aging

Mice were housed on a 12-h light/dark cycle and had *ad libitum* access to water and food. Eps8KO mice were backcrossed for ten generations into C57BL/6 mice and then used for the aging experiments. All other experiments were performed after an additional 8 generations of backcross. Both mice from heterozygous crosses (diet, metabolism, gene expression profile, everted sac) and homozygous F2N18 colonies (all remaining experiments) were used. Male mice were used unless otherwise indicated. For the diet experiments, mice were single housed at 10 weeks of age. After one week of habituation mice were placed either on a normal chow (Harlan Teklad Global 2018) or on a high fat diet (60% fat, D12492, Research Diets Inc.) and food intake and bodyweight were monitored for 10 weeks, twice weekly. All experiments were performed in accordance with the guidelines established in the IFOM-IEO Campus Principles of Laboratory Animal Care (directive 86/609/ECC).

### Analysis of the Metabolic Status

For glucose tolerance tests, mice were fasted overnight for 16 h and then injected intraperitoneally with 2 g/kg body weight glucose. For insulin tolerance test (ITT), mice were fasted for 5 h and then injected with 0.4 U/kg body weight (for 3 month old mice) or 0.5 U/kg body weight (for 6 and 12 month old mice) of human insulin (Eli Lilly) into the peritoneal cavity. Overnight fast was chosen for GTT since mice are night active and most of the food intake occurs during the night.; for ITT a 5 hrs fast was chosen because prolonged fasting causes the blood glucose values to drop too much after insulin administration. Blood glucose values were determined by an automatic glucose monitor (Glucotrend 2, Roche) and Accu-Chek active bands (Roche). Plasma parameters reported in Table S1 were measured as described in Text S1.

### Analysis of Intestinal Absorption

Everted intestinal sacs (1–1,5 cm length) were prepared from the upper part of the small intestine of WT and Eps8KO mice under constant oxygenation as described [37], [38]. Details are in Text S1.

For fecal dual isotope experiments, mice were starved overnight and then received a gastric bolus of 100 µl cornoil containing 1 µCi ^14^C-Oleic Acid and 1 µCi non-absorbable ^3^H-Sitostanol. Feces were collected for 4 days every 24 hrs. Samples were homogenized in 3 M KOH in 60% EtOH and incubated over night at 60°C under shaking to solubilize fatty acids [39]. Ten ml Hionic Fluor (Perkin Elmer) was added to 100 µl aliquots, left in the dark at room temperature for 4 h and then counted using a liquid scintillation counter. Absorption was calculated as:
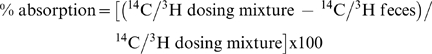



For plasma dual isotope experiments, mice were starved overnight, injected with 500 mg/kg Tyloxapol to inhibit plasma lipases and after 10 min given an intragastric bolus of 100 µl cornoil containing 2 µCi ^14^C-Oleic Acid and 2 µCi Triolein. Blood was sampled at the indicated time points and 10 µl were solubilized in 100 µl Isopropanol/Soluene-350 (1∶1) for 30 min. Ten µl 30% peroxide was added and samples were incubated overnight at 37°C in tightly capped vials. Samples were cooled to room temperature and 10 ml Hionic Fluor (Perkin Elmer) was added. Samples were left in the dark at room temperature for 4 h and then counted using a liquid scintillation counter.

*In vivo* intestinal permeability was measured as described in An et al., [40]. Intestinal transit time was measured as described in Friebe et al., [41]. Details are in Text S1.

### Analysis of Energy Expenditure

For the measurement of oxygen consumption and carbon dioxide production, mice were placed inside a climate chamber. Gas concentrations were measured by sucking compressed air through custom made metabolic chambers. Further details are in Text S1. Core body temperature (T_b_) and activity were monitored using implanted thermosensitive transmitters. For the determination of total daily energy expenditure (DEE) at room temperature mice were kept in metabolic cages with food and water ad libitum. Red mouse igloos (Plexx, NL) were offered as shelters. The measurement started in the afternoon of day one and was terminated in the morning of day three. This procedure enabled a recording of a complete data set of resting (photophase) and activity (scotophase) during a period of 24 consecutive hours. For the determination of the thermoneutral zone, metabolic rate was monitored within a wide range of ambient temperatures from 6 to 34°C. Details are described in Text S1.

### Histology and Ultrastructural Analysis

Intestinal segments from WT and Eps8KO mice were fixed in 10% buffered formalin, processed and embedded in paraffin. Five µm tissue sections were stained with H&E or Alcian Blue. Morphometric analysis was with the image analysis software Image-Pro Plus (version 4.5, Media Cybernetics, Silver Spring, USA). Ultrastructural analysis was essentially as in Croce et al. [10]. Briefly, intestinal pieces of 1×1 mm^2^, collected from the proximal duodenum of 4 WT and 4 Eps8KO male mice of 6 months of age, fed ad libidum on standard chow. Samples were first fixed in 2.5% glutaraldheyde for 2 h at room temperature, postfixed in osmium tetroxide for 2 h and then in uranyl acetate for another hour. Subsequently, samples were dehydrated through a graded ethanol series and propylene oxide and embedded in resin (Poly-Bed; Polysciences, Inc., Warrington, PA) overnight at 42°C and 2 days at 60°C. Ultrathin sections (50 nm) were collected, stained with uranyl acetate/lead citrate and observed under an electron microscope (model CM10 or Model G2 Tecnai; Philips, Eindhoven, The Netherlands). Morphometric analysis was performed arbitrarily in ten different regions per tissue block in which the intestinal tight junctions were well preserved as a read-out of the intactness of the tissue. A total length of 70 µm intestinal surface per animal was analyzed.

### Statistical Analyses

Means were compared between WT and Eps8KO mice using two-tailed Student's *t* test. Values in the text are means ± S.E.M. Differences were considered significant at *p*<0.05.

### Cell Lines and Cellular Biochemistry

Preparation of PEFs and macrophages, and conditions for the cultivation of 3T3-L1 and Caco-2 cells (obtained from ATCC-LGC) are in Text S1. Preparation of brush border membranes, determination of ROS, and IB analysis are also in Text S1.

### Gene Expression Analysis

Glass cDNA-chips were produced as recently described. A full description of the approximate 21.000 probes on the microarray is available in the GEO database (GPL4937). The expression data have been submitted to the GEO database (GSE14454).

For preparation of total RNA, individual organs were thawed in a buffer containing chaotropic salt (RLT buffer, Qiagen) and homogenized using a Polytron homogenizer. Total RNA from individual samples was obtained according to the manufacturer's protocols using RNeasy Midi kits (Qiagen). Microarray slides were hybridized and processed as described previously [42].

The TIGR Microarray Data Analysis System [TM4 [43], [44])] was used for normalization [MIDAS; [45]] and identification of genes with significant differential regulation [SAM, Significance Analysis of Microarrays; [46]]. Expression data were normalized by performing a total intensity normalization to transform the mean log_2_ ratio to zero. To eliminate low-quality array elements several filtering methods were applied. They included: background checking for both channel with a signal/noise threshold of 2.0, one bad tolerance policy parameter and flip dye consistency checking [47].

Validation was performed by quantitative PCR using Roche chemistry on selected genes.

### Gene Expression Meta Analysis

Microarray gene expression data of Pohjanvirta et al. [22] (GSE9121, Affymetrix RAT230 2.0 chip), and Bauer et al. [23] (GSE858, custom two channels cDNA microarray) were downloaded from Gene Expression Omnibus (GEO, www.ncbi.nlm.nih.gov/geo/). Normalized data were log2 transformed and analyzed using GeneSpring GX 7.3 (Agilent Technologies, USA). Statistical analysis was performed using a two-samples parametric Welch's t-test (variance not assumed equal) followed by multiple testing correction (Benjiamini and Hochberg False Discovery Rate). All genes with a *P*-value less than 0.05 were considered to be significantly regulated. In the Bauer et al. study, a direct design for two colors cDNA microarray experiment was employed (i.e. the treated versus control condition were analyzed directly on the same chip) and the sole gene expression profile of control mice, as in the Pohjanvirta et al. study, was not available. Therefore, in order to analyze this dataset consistently with the analysis of Pohjanvirta et al. (i.e. two-samples Welch's t-test with multiple test correction), we compared the 48 h starved group with the 48 h sugar-supplemented group.

## Supporting Information

Text S1 Supplemental experimental procedures for Plasma parameters, Everted sac experiments, *In vivo* Intestinal Permeability, Intestinal transit time, Feces energy content, Open respiratory system, Body temperature and activity, Determination of the thermoneutral zone (TNZ) and basal metabolic rate (BMR), Soxhlet analysis, Primary embryonic fibroblasts, 3T3-L1, Caco-2, Macrophages, Determination of Reactive Oxygen Species (ROS), Immunoblotting, Preparation of intestinal brush-border membranes.
(0.07 MB DOC)

Figure S1 Normal intestinal permeability during everted sac assays *in vitro*. A–C. Bar graphs depicting the uptake of 3H-Mannitol during everted sac uptake assays in sacs obtained from WT and Eps8KO mice. Uptake was also monitored after addition of cold competitors (grey bars), specific for each assay: A, 40 mM Oleic Acid (OA, n = 6); B, 20 mM Sarcosyl-glycine (SG, n = 4); and C, 100 mM Methyl-D-glucopyranoside (MDG, n = 4). Values are expressed as mean ± SEM; significance was assessed using 2-tailed student's t-test and no difference was found between genotypes.
(0.15 MB PDF)

Figure S2 Increased lifespan in both male and female Eps8KO mice, and ROS production during FcγR-mediated internalization in macrophages. A–B. Kaplan-Meyer survival curves depicting increased survival of: A, male; B female Eps8KO and WT mice. The mean and median survival is significantly increased both in male and female Eps8KO mice (P = 0.02 and P = 0.009, respectively, independent of the sex). C. Bar graphs depicting relative fluorescence of DCFDA after FcγR-mediated internalization in wild-type (WT) or Eps8KO (KO) peritoneal exudate macrophages. After addition of the internalization stimulating immune-complex, cells were kept on ice (4°C, negative control) or shifted to 37°C to allow for internalization and subsequent ROS production. No significant difference was observed between genotypes. The experiment was performed in duplicate (at 4°C) or triplicate (at 37°C) with n = 3 per genotype.
(0.20 MB PDF)

Figure S3 Eps8 is enriched in the intestinal brush border membrane fraction. Left, schematics of intestinal brush border membrane preparation. Right, immunoblot analysis of an intestinal brush border membrane preparation. Individual fractions (equal amount of proteins were loaded) were blotted for Ezrin, a *bona fide* brush border membrane protein, for Sos1, a cytosolic protein (as a negative control) and for Eps8, as indicated. S0 indicates the starting homogenate, S1–S4 the supernatants and P1–P4 the pellets of the subsequent purification steps. P4 represents the final brush border membrane fraction. The enrichment (per mg of loaded proteins) is also given, as assessed by densitometric scans of the immunoblots.
(0.24 MB PDF)

Table S1 Blood parameters. Blood parameters of 6 month-old Eps8KO (KO) and wild-type (WT) mice, fed for 10 weeks on a high fat diet (HFD, 60% fat of caloric intake) or on normal chow (ND). F indicates a fed state and S a starved state (overnight fast) before blood sampling. Values are reported as mean ± SEM (n  =  number of mice per experiment). Significance was assessed using two-tailed student's t-test: *, P<0.05; **, P<0.01; ***; P<0.005.
(0.06 MB DOC)

Table S2 Expression profile of Eps8KO liver. Genes that were differentially expressed in Eps8KO liver respect to wild-type mice by gene-chip analysis are shown. For each gene we show: the common name (Gene), the accession number, the category derived from the Gene Ontology term, the known function, the level of differential expression in the gene-chip analysis (Chip fold change), and the validation by QPCR (QPCR fold change). ND, not done.
(0.06 MB DOC)

Table S3 Meta-analysis of Eps8KO regulated genes. Meta analysis of genes differentially expressed in the liver of the Eps8KO/WT comparison in two independent datasets of CR mice. In the study by Pohjanvirta et al. 2008, two conditions of starvation (4 days and 10 days) were used, and both are reported in the comparison. In the study by Bauer et al. 2004, several conditions were used and we report the longest one (48 h) in the comparison. These data were used to create panel B of Fig. 5. In that panel, the study by Pohjanvirta et al. 2008 is referred as CR-II, and the study by Bauer et al. 2004 as CR-III. In that panel (Fig. 5B), we also computed the data from another study by Dhabhi et al. 2004 (referred to in the panel as CR-I). In the case of the study by Dhabhi et al. 2004, however, the raw data of the dataset are not available. We could not therefore perform a metanalysis, and simply used the gene list of differentially expressed genes as the authors reported it in their publication. In this table, each gene differentially expressed in the Eps8KO/WT comparison (column “Gene”) is followed by the chip fold change in the Eps8KO/WT comparison (in all cases P<0.05), and by the metanalyzed data from Pohjanvirta et al. 2008 and Bauer et al. 2004 indicating the fold change and the P-value (Welch's t-test, Hochberg and Benjamini correction). See details in Materials and Methods. NP, genes not present on the microarray platform used in the indicated studies.
(0.06 MB DOC)
