# Supplementary material for: Loss of the Actin Remodeler Eps8 Causes Intestinal Defects and Improved Metabolic Status in Mice
Source: PLoS One. 2010 Mar 2;5(3):e9468. doi: 10.1371/journal.pone.0009468 (PMC2830459; doi:10.1371/journal.pone.0009468)
Supplement: Text S1 — Supplemental experimental procedures for Plasma parameters, Everted sac experiments, In vivo Intestinal Permeability, Intestinal transit time, Feces energy content, Open respiratory system, Body temperature and activity, Determination of the thermoneutral zone (TNZ) and basal metabolic rate (BMR), Soxhlet analysis, Primary embryonic fibroblasts, 3T3-L1, Caco-2, Macrophages, Determination of Reactive Oxygen Species (ROS), Immunoblotting, Preparation of intestinal brush-border membranes. (0.07 MB DOC) [file pone.0009468.s001.doc]

**SUPPLEMENTAL EXPERIMENTAL PROCEDURES**

**Plasma parameters**

Plasma was prepared from blood samples collected from the retro-orbital sinus of mice (under ether narcosis) non-fasted or fasted overnight for 16 h. The blood samples were immediately centrifuged for 10 min at 10,000 rpm and then the plasma was removed and stored at -80 °C until used. Plasma insulin levels were measured by ELISA, using mouse insulin as standard (Crystal Chem). Plasma leptin was measured by ELISA (Crystal Chem). Adiponectin (ACRP30) and resistin plasma levels were measured by ELISA (BioVendor, GmbH). Alternatively, blood samples were collected in Lithium-Heparin coated tubes from the retro-orbital sinus of mice, which were under ether narcosis. The blood samples were left to stand for 2 h at room-temperature after which they were centrifuged for 10 min at 7000 U/min. Following centrifugation, the plasma was removed and stored at -20 °C until used. Clinical chemical parameters were measured by the clinical-chemistry laboratory of the German Mouse Clinic (GMC) using an automatic analyzer AU 400 (Olympus) and adapted test kits from Olympus (Hamburg, Germany) and in the case of FFA Wako Chemicals GmbH (Neuss, Germany).

**Everted sac experiments**

Six everted intestinal sacs per mouse (1-1.5 cm in length) were prepared from the upper part of the small intestine of WT and Eps8KO mice in Krebs-Ringer-HEPES under constant oxygenation as described [1,2]. After 5 min of preincubation at 37 °C, 3 sacs per condition were incubated with either ^14^C-Oleic Acid (OA) (0.1 μCi /ml plus 40 μM cold OA bound to fatty-acid-free BSA ± 40 mM BSA-bound OA, for 10 min), or ^14^C-Glycyl-Sarcosine (GlySar) (0.1 μCi/ml ± 10 mM GlySar, for 10 min) or ^14^C-Methyl-D-glycopyranoside (MDG) (0.1 μCi /ml ± 30 mM MDG, for 2 min). ^3^H-Mannitol (0.1 μCi/ml) was included in all assays to correct for the adhering water layer. Nonspecific binding was determined by addition of excess cold competitor as indicated. After uptake, sacs were immediately washed in ice-cold KRH, dried on a piece of paper towel, weighed and solubilized in 1 ml Soluene-350 (Perkin Elmer) by shaking at 50° C over night. Samples were cooled to room temperature and mixed with 10 ml Hionic Fluor Scintillation Cocktail (Perkin Elmer), left at room temperature in the dark for 4 h, and counted using a liquid scintillation counter.

**In vivo Intestinal Permeability**

Intestinal permeability was measured as described in An et al., [3]. Briefly, WT and Eps8KO mice received a bolus of FITC-dextran (600 mg/kg body weight; 4 kD; Sigma-Aldrich) by gavage. Blood was collected 4 h later by retro-orbital bleeding. The serum concentration of FITC-dextran was determined using a fluorimeter (PerkinElmer Life Sciences) with excitation wavelength at 490 nm and an emission wavelength of 530 nm. Serial-diluted FITC-dextran was used to generate a standard curve.

**Intestinal transit time**

Intestinal transit time was measured as described in Friebe et al., [4]. Briefly, carmine (100 μl; 3 mg of carmine in water) was orally administered to each mouse. Mice were returned to individual cages and placed on a white sheet of paper. The time taken for excretion of the first colored feces was recorded.

**Feces energy content**

Feces were collected from single housed mice twice weekly and weighted. Samples of the feces (~1 g) were dried at 60°C for two days, homogenized in a coffee grinder and squeezed to a pill for determination of energy content in a bomb calorimeter (IKA Calorimeter C7000) based on dry measurement principle.

**Open respiratory system (Indirect calorimetry)**

For the measurement of oxygen consumption and carbon dioxide production mice were placed inside a climate chamber (WK11, Weiss, Germany). Gas concentrations were measured by sucking compressed air through custom made metabolic chambers (volume 5 L) with a flow rate of 50 L*h^-1^ (measured by electronic mass flow meters (Tylan 260, Tylan, Germany)). Ambient temperature in the climate chamber and within the metabolic cages (T_a_) was continuously monitored. The animal air was dried by cooling traps (ECP1000 M&C, Germany) and led through a pneumatic switch unit containing filters (Schleicher&Schuell, Germany), pumps (Schego, Germany) and magnetic valves (Buerkert, Germany). The magnetic valve system allowed the measurement of up to four mice simultaneously. A fifth channel was used for zero reference checks. A computer software (Akrobit, Germany) triggered the valves at one min intervals via an interface. The O2 and CO2 content of the individual animal chambers were analyzed by O2 and CO2 analyzers (Magnos106, Uras14, ABB, Germany). Oxygen consumption (VO2) and carbon dioxide production (VCO2) were calculated according to: VO2 [ml O2 h^-1^] = (dVol% O2) * Flow rate [L h^-1^] * 10 and VCO2 [ml CO2 h^-1^] = (dVol% CO2) * Flow rate [L h^-1^] * 10 [see [5]].

**Body temperature and activity**

Core body temperature (T_b_) and activity were monitored using implanted thermosensitive transmitters (VitalView Series 3000, MiniMitter, USA). Prior to implantation transmitters (model XM-FH) were calibrated in a water bath (K15, Haake, Germany) at 32 °C and 39 °C for determination of transmitter coefficients. For the intraperitoneal implantation of the transmitters, mice were anaesthetized with ketamin (75 mg kg^-1^; Pharmacia GmbH, Germany) and xylacine (2 mg kg^-1^; Bayer, Germany). Peritoneum and skin were sutured with resorbable thread (Marlin, Catgut GmbH, Germany). For recovery, mice were kept for one week under *ad libitum* conditions. The receiver panel (RTA-500, MiniMitter, USA) was placed inside the climate chamber below the metabolic cages. Signals were interfaced (Dataport DP24, MiniMitter, USA) and stored on a computer system (VitalView, MiniMitter, USA) in one min intervals. In the diet experiments, temperature was determined using a rectal probe.

**Determination of the thermoneutral zone (TNZ) and basal metabolic rate (BMR)**

The thermoneutral zone is defined as the range of T_a_ under which basal metabolic rate (BMR) and heat production (HP) are sufficient to maintain normothermy. The lower and upper critical temperatures (T_lc_ and T_uc_) are defined as the temperatures where energy expenditure increases again. For the determination of the TNZ, mice were kept in metabolic cages without access to food or water and exposed to different T_a_ ranging from 6 °C to 34 °C in eight temperature steps of 30 to 60 min each. After an adaptation phase of one h, data collection started at housing temperature of 24±1 °C. The mean value of three consecutive registrations of oxygen consumption of resting mice represented the resting metabolic rate (RMR) at each T_a_ (see [6]). The basal metabolic rate (BMR) is defined as the lowest oxygen consumption measured within the TNZ. All metabolic measurements for determination of TNZ were conducted during daytime between 8:30 am and 5:30 pm (CET).

**Soxhlet analysis**

For body composition analysis animals were killed, weighed and the gastrointestinal tract was removed. The carcasses were then dried to constant weight at 60°C. Desiccated carcasses were weighed again to calculate water content. Fat mass was determined by extracting the lipids from the carcass using a refluxing Soxhlet apparatus with petrolether as solvent for 16 hours. Post extraction dry mass was determined and lean mass was calculated as carcass weight minus fat mass.

**Primary embryonic fibroblasts**

Primary embryonic fibroblasts (PEFs) were derived from WT and Eps8KO mice according to standard procedures and cultured in Dulbecco’s modified Eagle’s medium (DMEM) containing 10% fetal bovine serum (FBS). For the analysis of insulin signaling, PEFs were starved overnight with DMEM containing 0.5% FBS, then treated for the indicated time points with insulin 10 μg/ml, followed by lysis in RIPA buffer containing 10 mM Na_2_HPO_4_, 10 mM NaH_2_PO_4_, pH 7.4, 100 mM NaCl, 1% Triton X-100, 0.1% SDS, 5 mM EDTA, and phosphatase and protease inhibitors **(**Roche 100x).

**3T3-L1**

3T3-L1 pre-adipocytes were obtained from ATCC-LGC (# CL-173) and cultured in DMEM containing 10% calf serum until confluent and maintained for an additional 2 days (day 0). Differentiation was induced on day 0 by the addition of 250 μM isobutyl-methylxantine, 250 nM dexamethasone, 5 μg/ml of insulin and 10% FBS in DMEM. After 48 h (day 2) the medium was replaced with DMEM containing 5 μg/ml of insulin and 10% FBS. After day 4, the cells were fed every second day with 10% FBS in DMEM. Stealth oligos were delivered to 3T3-L1 adipocytes differentiated for 4-5 days, by electroporation. Cells were trypsinized, washed twice with PBS, resuspended in 0.8 ml PBS. 2 nM oligos were electroporated per five million cells using 160mV and 950 mFarrad. After electroporation cells were replated at twice the original density to reach confluence despite cell death. Cells were used for experiments 2-3 days after electroporation. For the analysis of insulin signaling, cells were washed twice with DMEM and starved for 12 h, and then treated with insulin (3 ng/ml) for different time points.

**Caco-2**

Caco-2 cells were obtained from ATCC-LGC (# HTB-37) and cultured in DMEM with 20% South American serum, 1% glutamine, 1% non-essential amino acids and 1% Sodium Pyruvate. Cells were transfected using calcium phosphate at 85% confluency with plasmids expressing Eps8-GFP or GFP alone under a chicken β-actin promoter. Confluent cells were split and plated on collagen-coated coverslips (50 μg/ml) at the concentration of 6.6 x 10^5^ cells/cm^2^. Cells were allowed to differentiate for 7-15 days, fixed with 4% paraformaldehyde and stained with phalloidin-TRITC to visualize F-actin.

**Macrophages**

Murine peritoneal exudate macrophages (PEM) were harvested 72 h after intraperitoneal injection of 3% thioglycolate. PEMs were washed twice with chilled PBS and immediately used for the functional assays. The number of macrophages in the preparation was 90-95% as determined by May Grundwald-Giemsa staining of cytospins.

**Determination of Reactive Oxygen Species (ROS)**

Primary embryonic fibroblasts were incubated for 45 min in complete media with 4-12 μM DCFDA (Invitrogen), washed and subsequently analyzed by flow cytometry for fluorescence intensity (Facscalibur).

FcγR-mediated internalization of immune complexes leading to oxidative burst in the phagosomal vacuole of peritoneal exudates macrophages was monitored using the Fc OxyBURST® reagent from Molecular Probes. The reagent consists of insoluble BSA-anti-BSA immune complexes in which the BSA is covalently labeled with dichlorodihydrofluorescein (H_2_DCF). Oxidation of the H_2_DCF to DCF in the phagosomal vesicle produces green fluorescence that can be monitored by flow cytometry. For this assay, freshly made PEMs were resuspended in Krebs' Ringer's PBS (KRP buffer) with 1.0 mM Ca^2+^, 1.5 mM Mg^2+^ and 5.5 mM glucose and OxyBurst reagent added, followed by incubation for 30 minutes at 37°C or on ice. Fluorescence related to oxidation of Fc OxyBurst was assayed by flow cytometry using a FACSCalibur.

**Immunoblotting**

Cells or tissues were lysed in RIPA buffer containing 10 mM Na_2_HPO_4_, 10 mM NaH_2_PO_4_, pH 7.4, 100 mM NaCl, 1% Triton X-100, 0.1% SDS, 5 mM EDTA , and phosphatase and protease inhibitors **(**Roche 100x). Protein concentration was determined using BCA (Pierce). After transfer onto Nylon-Membranes (Amersham), nonspecific binding was blocked in Tris-buffered saline (50 mM Tris pH 8.0, 150 mM NaCl) containing 0.05 % Tween-20 and 5% BSA, followed by immunoblotting with the indicated antibodies. Primary antibodies were detected with HRP-coupled secondary antibodies and enhanced chemiluminescence (ECL, Amersham). The following antibodies were employed: anti-Akt, anti-phospho-Akt (Ser473), anti-phospho-ERK1/2, anti-JNK, anti-phospho-JNK (all from Cell Signalling), anti-ERK1/2 (Sigma), anti-Eps8 (BD Biosciences), anti-Vinculin (Sigma) and anti-PPARγ (Santa Cruz).

**Preparation of intestinal brush-border membranes**

Microvillar membranes were prepared from uncultured intestinal mucosal scrapings of fasted animals by the divalent cation precipitation method [7]. Briefly, scrapings (100 mg) were homogenized at 4° C in 2 mM Tris HCl, 10 mM mannitol, pH 7.1, using a manually operated Potter-Elvehjem homogenizer (total volume 1.0 ml). The homogenate was cleared by centrifugation at 500 g for 10 min and MgCl_2_ was added to a final concentration of 10 mM (S0). After incubation on ice for 15 min with occasional stirring, the preparation was centrifuged at 1500 g for 12 min to pellet intracellular and basolateral membranes (P1). The supernatant (S1) was centrifuged at 15,000 g for 12 min and the pellet was resuspended in 0.5 ml mannitol-Tris-MgCl_2_ added as before to a final 10mM concentration (P2). The preparation was centrifuged at 2,200 g for 12 min and finally the supernatant (S3) was centrifuged at 15,000g for 12 min to obtain a pellet of microvillar membrane vesicles (P4).

**REFERENCES TO SUPPLEMENTAL INFORMATION**

1. Arellano C, Philibert C, Lacombe O, Woodley J, Houin G (2004) Liquid chromatographic-mass spectrometric method to assess cytochrome P450-mediated metabolism of testosterone by rat everted gut sacs. J Chromatogr B Analyt Technol Biomed Life Sci 807: 263-270.

2. Sato T, Mushiake S, Kato Y, Sato K, Sato M, et al. (2007) The Rab8 GTPase regulates apical protein localization in intestinal cells. Nature 448: 366-369.

3. An G, Wei B, Xia B, McDaniel JM, Ju T, et al. (2007) Increased susceptibility to colitis and colorectal tumors in mice lacking core 3-derived O-glycans. J Exp Med 204: 1417-1429.

4. Friebe A, Mergia E, Dangel O, Lange A, Koesling D (2007) Fatal gastrointestinal obstruction and hypertension in mice lacking nitric oxide-sensitive guanylyl cyclase. Proc Natl Acad Sci U S A 104: 7699-7704.

5. Heldmaier G, Steinlechner S (1981) Seasonal control of energy requirements for thermoregulation in the Djungarian Hamster (Phodopus sungorus), living in natural photoperiod. J Comp Physiol 142: 429-437.

6. Meyer CW, Klingenspor M, Rozman J, Heldmaier G (2004) Gene or size: metabolic rate and body temperature in obese growth hormone-deficient dwarf mice. Obes Res 12: 1509-1518.

7. Booth AG, Kenny AJ (1974) A rapid method for the preparation of microvilli from rabbit kidney. Biochem J 142: 575-581.
