# Supplementary figures and images for: Loss of the Actin Remodeler Eps8 Causes Intestinal Defects and Improved Metabolic Status in Mice
Source: PLoS One. 2010 Mar 2;5(3):e9468. doi: 10.1371/journal.pone.0009468 (PMC2830459; doi:10.1371/journal.pone.0009468)

**A**

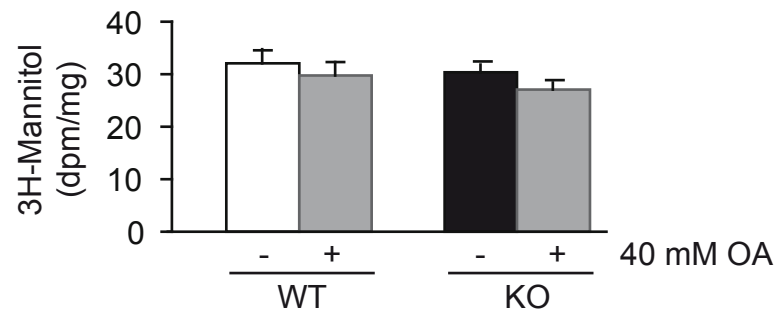

**B**

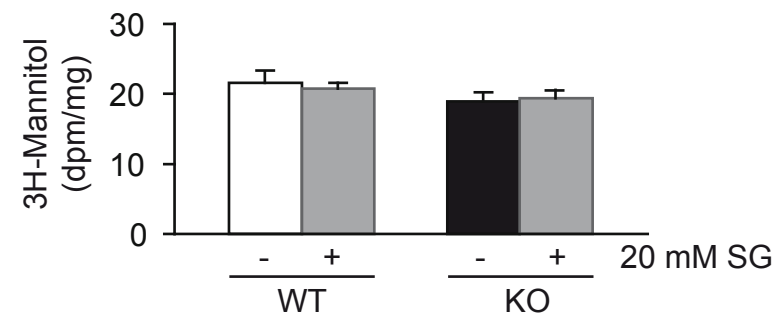

**C**

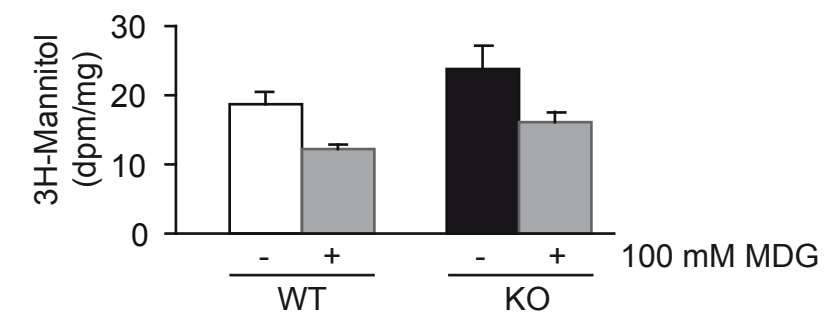

Figure S1

Supplement: Figure S1 — Normal intestinal permeability during everted sac assays in vitro. A–C. Bar graphs depicting the uptake of 3H-Mannitol during everted sac uptake assays in sacs obtained from WT and Eps8KO mice. Uptake was also monitored after addition of cold competitors (grey bars), specific for each assay: A, 40 mM Oleic Acid (OA, n = 6); B, 20 mM Sarcosyl-glycine (SG, n = 4); and C, 100 mM Methyl-D-glucopyranoside (MDG, n = 4). Values are expressed as mean ± SEM; significance was assessed using 2-tailed student's t-test and no difference was found between genotypes. (0.15 MB PDF) [file pone.0009468.s002.pdf]

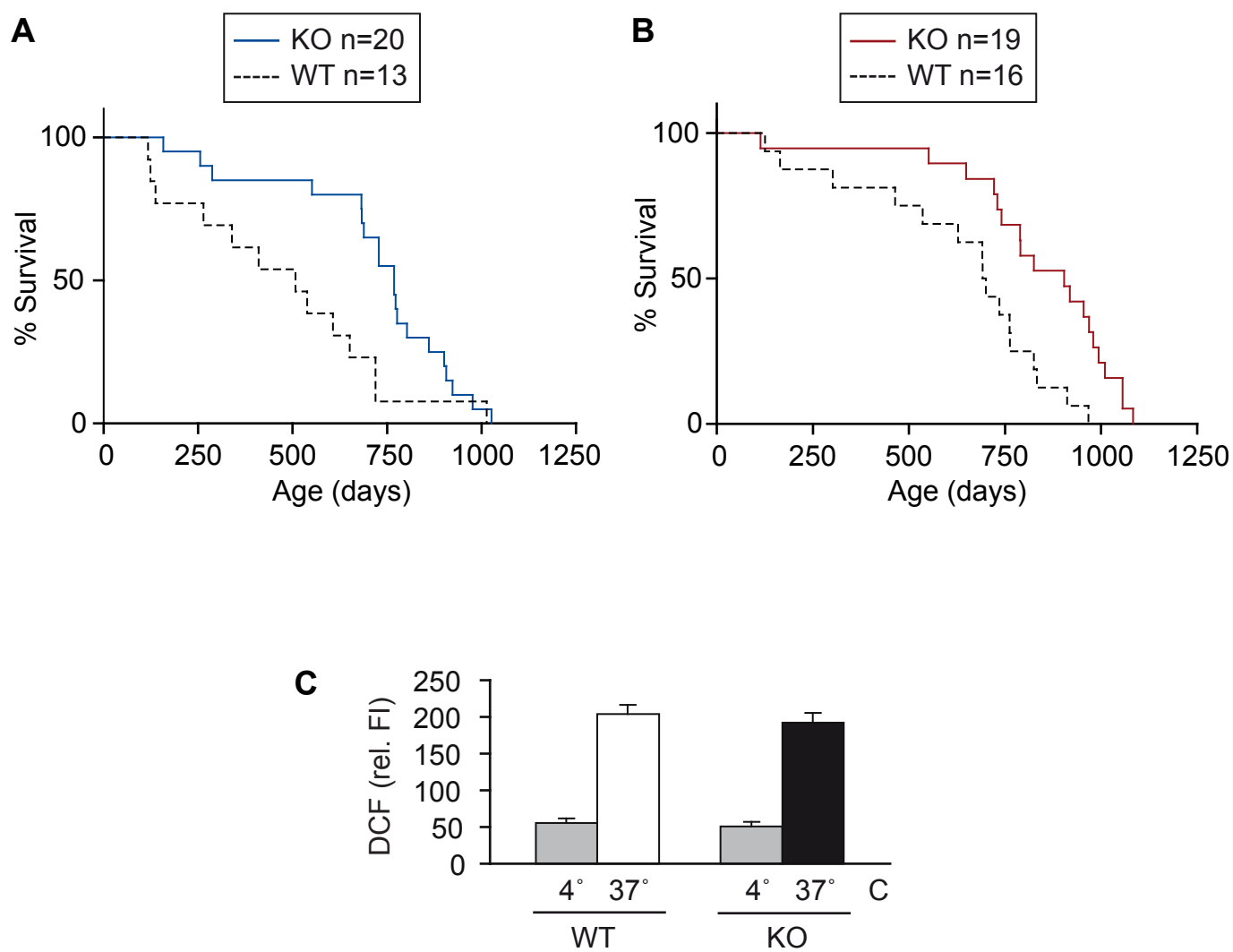

Figure S2

Supplement: Figure S2 — Increased lifespan in both male and female Eps8KO mice, and ROS production during FcγR-mediated internalization in macrophages. A–B. Kaplan-Meyer survival curves depicting increased survival of: A, male; B female Eps8KO and WT mice. The mean and median survival is significantly increased both in male and female Eps8KO mice (P = 0.02 and P = 0.009, respectively, independent of the sex). C. Bar graphs depicting relative fluorescence of DCFDA after FcγR-mediated internalization in wild-type (WT) or Eps8KO (KO) peritoneal exudate macrophages. After addition of the internalization stimulating immune-complex, cells were kept on ice (4°C, negative control) or shifted to 37°C to allow for internalization and subsequent ROS production. No significant difference was observed between genotypes. The experiment was performed in duplicate (at 4°C) or triplicate (at 37°C) with n = 3 per genotype. (0.20 MB PDF) [file pone.0009468.s003.pdf]

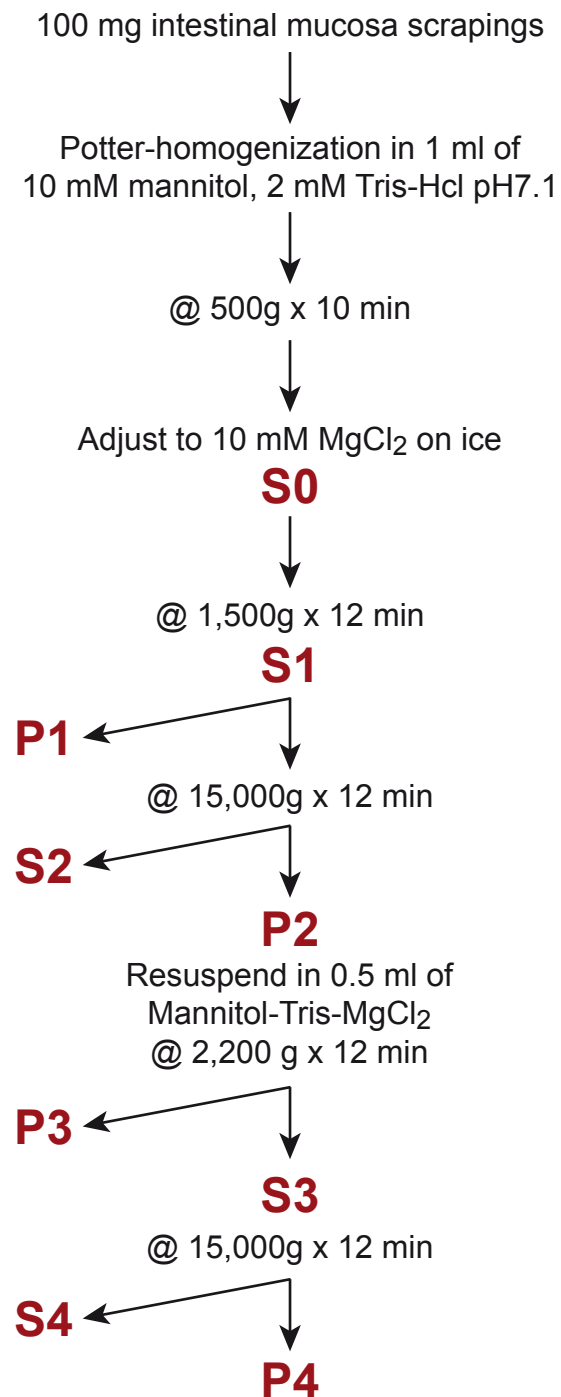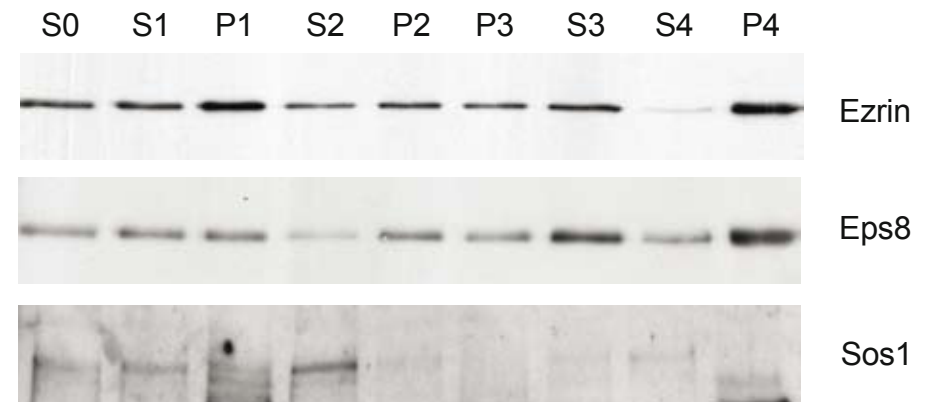

#### Enrichment P4/S0 (per mg of protein)

Eps8: 2.7

Ezrin: 1.5

Sos1: 0.1

Figure S3

Supplement: Figure S3 — Eps8 is enriched in the intestinal brush border membrane fraction. Left, schematics of intestinal brush border membrane preparation. Right, immunoblot analysis of an intestinal brush border membrane preparation. Individual fractions (equal amount of proteins were loaded) were blotted for Ezrin, a bona fide brush border membrane protein, for Sos1, a cytosolic protein (as a negative control) and for Eps8, as indicated. S0 indicates the starting homogenate, S1–S4 the supernatants and P1–P4 the pellets of the subsequent purification steps. P4 represents the final brush border membrane fraction. The enrichment (per mg of loaded proteins) is also given, as assessed by densitometric scans of the immunoblots. (0.24 MB PDF) [file pone.0009468.s004.pdf]
