# Supplementary material for: Loss of the Actin Remodeler Eps8 Causes Intestinal Defects and Improved Metabolic Status in Mice
Source: PLoS One. 2010 Mar 2;5(3):e9468. doi: 10.1371/journal.pone.0009468 (PMC2830459; doi:10.1371/journal.pone.0009468)
Supplement: Table S1 — Blood parameters. Blood parameters of 6 month-old Eps8KO (KO) and wild-type (WT) mice, fed for 10 weeks on a high fat diet (HFD, 60% fat of caloric intake) or on normal chow (ND). F indicates a fed state and S a starved state (overnight fast) before blood sampling. Values are reported as mean ± SEM (n = number of mice per experiment). Significance was assessed using two-tailed student's t-test: *, P<0.05; **, P<0.01; ***; P<0.005. (0.06 MB DOC) [file pone.0009468.s005.doc]

**Table S1: Blood parameters in Eps8KO mice.**

| **Parameter and fed state**  **(F = fed; S= starved)** | | **Strain and type of diet** | | | | **Significance (*P*)** | | | |
| --- | --- | --- | --- | --- | --- | --- | --- | --- | --- |
|  |  | **WT ND** | **KO ND** | **WT HFD** | **KO HFD** | **WT-ND/**  **KO-ND** | **WT-HFD/**  **KO-HFD** | **WT-ND/**  **WT-HFD** | **KO-ND/**  **KO-HFD** |
| **Bodyweight (g)** | F | 29.7±0.8 (15) | 27.4±1.6 (13) | 46.4±0.4 (14) | 37.7±0.7 (13) | * | *** | * | *** |
| **Insulin (ng/ml)** | S | 2.0±0.2 (14) | 1.1±0.1 (12) | 5.2±0.3 (11) | 3.2±0.5 (11) | *** | *** | *** | *** |
| **Leptin (ng/ml)** | S | 4.1±0.7 (8) | 2.3±0.3 (8) | 37.1±1.5 (8) | 28.8±2.9 (10) | * | * | *** | *** |
| **Adiponektin (ng/ml)** | S | 2.7±0.2 (9) | 2.3±0.3 (9) | 2.0±0.2 (9) | 2.4±0.2 (9) |  |  | * |  |
| **Glucose (mg/dl)** | F | 160.4±7.2 (7) | 155.1±5.6 (9) | 206.8±12.2 (14) | 178.0±14.2 (15) |  |  | * |  |
| **Glucose (mg/dl)** | S | 131.8±3.1 (9) | 122.6±4.1 (8) | 177.1±4.4 (8) | 141.0±6.9 (9) | *** | *** | *** | * |
| **Cholesterol**  **(mg/dl)** | F | 148.3±11.9 (7) | 147.6±3.2 (9) | 239.6±9.5 (14) | 202.1±13.1 (15) |  | * | *** | *** |
|  | S | 123.8±4.5 (8) | 101.2±4.7 (8) | n.d. | n.d. | *** |  |  |  |
| **LDL (mg/dl)** | F | 23.7±1.7 (7) | 22.1±1.0 (9) | 40.8±1.4 (14) | 33.3±2.0 (15) |  | ** | *** | *** |
|  | S | 10.8±1.3 (8) | 5.8±0.9 (8) | n.d. | n.d. | ** |  |  |  |
| **HDL (mg/dl)** | F | 105.1±8.6 (7) | 105.2±1.8 (9) | 168.0±7.4 (14) | 144.4±9.3 (15) |  |  | *** | *** |
|  | S | 99.7±3.2 (8) | 82.9±3.3 (8) | n.d. | n.d. | *** |  |  |  |
| **FFA (mmol/l)** | F | 0.91±0.03 (7) | 0.97±0.05 (9) | 1.18±0.09 (14) | 1.14±0.13 (15) |  |  |  |  |
|  | S | 1.23±0.08 (8) | 1.22±0.06 (8) | n.d. | n.d. |  |  |  |  |
| **Triglycerides (mg/dl)** | F | 145.0±9.5 (7) | 140.5±15.7 (9) | 128.1±21.8 (14) | 94.6±6.6 (15) |  |  |  | *** |
|  | S | 84.3±7.5 (8) | 85.0±6.1 (8) | n.d. | n.d. |  |  |  |  |
