# Supplementary material for: Loss of the Actin Remodeler Eps8 Causes Intestinal Defects and Improved Metabolic Status in Mice
Source: PLoS One. 2010 Mar 2;5(3):e9468. doi: 10.1371/journal.pone.0009468 (PMC2830459; doi:10.1371/journal.pone.0009468)
Supplement: Table S2 — Expression profile of Eps8KO liver. Genes that were differentially expressed in Eps8KO liver respect to wild-type mice by gene-chip analysis are shown. For each gene we show: the common name (Gene), the accession number, the category derived from the Gene Ontology term, the known function, the level of differential expression in the gene-chip analysis (Chip fold change), and the validation by QPCR (QPCR fold change). ND, not done. (0.06 MB DOC) [file pone.0009468.s006.doc]

| **Gene** | **Accession**  **Number** | **Category** | **Function** | **Chip**  **fold change** | **QPCR**  **fold change** |
| --- | --- | --- | --- | --- | --- |
| Car3 | NM_007606 | Metabolism | Carbonic anhydrase | 3.6 | 2.6 |
| Inmt | NM_009349 | Metabolism | Transferase | 3.6 | ND |
| Cat | NM_009804 | Metabolism | Catalase | 1.9 | ND |
| Ass1 | NM_007494 | Metabolism | Urea cycle | 1.6 | 1.7 |
| Pah | NM_008777 | Metabolism | Fatty acid biosynthesis | 1.6 | 1.7 |
| Scd1 | NM_009127 | Metabolism | Fatty acid biosynthesis | 1.6 | 1.7 |
| Fga | NM_010196 | Stress response | Blood clotting | -1.6 | ND |
| Hspa8 | NM_031165 | Stress response | Chaperone | -1.6 | ND |
| Fn1 | NM_010233 | Inflammation | Acute phase response | -1.7 | ND |
| Ambp | NM_007443 | Inflammation | Antioxidant | -1.8 | ND |
| Ly6e | NM_008529 | Inflammation | Immune response | -1.8 | -1.7 |
| C3 | NM_009778 | Inflammation | Immune response | -1.9 | ND |
| Ldha | NM_010699 | Metabolism | Citric acid cycle | -2.2 | -1.7 |
| Mbl1 | NM_010775 | Inflammation | Acute phase response | -2.4 | -1.5 |
| Cp | NM_007752 | Inflammation | Antioxidant | -2.5 | ND |
| Armet | NM_029103 | Stress response | ER Chaperone | -3.1 | -2.2 |
| Serpina3k | NM_009252 | Inflammation | Endopeptidase inhibitor | -3.3 | ND |
| Mup1 | NM_031188 | Metabolism | Transporter | -4.2 | -2.6 |
| Hpx | NM_017371 | Inflammation | Antioxidant | -5.0 | ND |
| Orm1 | NM_008768 | Inflammation | Acute phase response | -6.1 | ND |
