# Supplementary material for: Loss of the Actin Remodeler Eps8 Causes Intestinal Defects and Improved Metabolic Status in Mice
Source: PLoS One. 2010 Mar 2;5(3):e9468. doi: 10.1371/journal.pone.0009468 (PMC2830459; doi:10.1371/journal.pone.0009468)
Supplement: Table S3 — Meta-analysis of Eps8KO regulated genes. Meta analysis of genes differentially expressed in the liver of the Eps8KO/WT comparison in two independent datasets of CR mice. In the study by Pohjanvirta et al. 2008, two conditions of starvation (4 days and 10 days) were used, and both are reported in the comparison. In the study by Bauer et al. 2004, several conditions were used and we report the longest one (48 h) in the comparison. These data were used to create panel B of Fig. 5. In that panel, the study by Pohjanvirta et al. 2008 is referred as CR-II, and the study by Bauer et al. 2004 as CR-III. In that panel (Fig. 5B), we also computed the data from another study by Dhabhi et al. 2004 (referred to in the panel as CR-I). In the case of the study by Dhabhi et al. 2004, however, the raw data of the dataset are not available. We could not therefore perform a metanalysis, and simply used the gene list of differentially expressed genes as the authors reported it in their publication. In this table, each gene differentially expressed in the Eps8KO/WT comparison (column “Gene”) is followed by the chip fold change in the Eps8KO/WT comparison (in all cases P<0.05), and by the metanalyzed data from Pohjanvirta et al. 2008 and Bauer et al. 2004 indicating the fold change and the P-value (Welch's t-test, Hochberg and Benjamini correction). See details in Materials and Methods. NP, genes not present on the microarray platform used in the indicated studies. (0.06 MB DOC) [file pone.0009468.s007.doc]

| **Gene** | **Eps8**  **KO/wt** | **Pohjanvirta et al.**  **(4 days)** | | **Pohjanvirta et al.**  **(10 days)** | | **Bauer et al.**  **(2 days)** | |
| --- | --- | --- | --- | --- | --- | --- | --- |
|  | **Fold** | **Fold** | **P** | **Fold** | **P** | **Fold** | **P** |
| Car3 | 3.6 | -1.3 | 0.073 | -3.0 | 0.033 | -17.2 | <0.001 |
| Inmt | 3.6 | 2.9 | 0.039 | -1.2 | 0.383 | NP | NP |
| Cat | 1.9 | -1.1 | 0.177 | -1.1 | 0.057 | -1.5 | <0.001 |
| Ass1 | 1.6 | 1.1 | 0.059 | 1.2 | 0.024 | 1.2 | 0.126 |
| Pah | 1.6 | 1.2 | 0.008 | 1.2 | 0.024 | -1.1 | 0.594 |
| Scd1 | 1.6 | -104 | 0.001 | -121 | <0.001 | -13.7 | <0.001 |
| Fga | -1.6 | -1.1 | 0.168 | -1.2 | 0.007 | -1.4 | <0.001 |
| Hspa8 | -1.6 | 1.5 | 0.004 | 1.5 | 0.007 | 1.5 | <0.001 |
| Fn1 | -1.7 | -1.3 | 0.014 | -1.3 | 0.001 | -1.5 | <0.001 |
| Ly6e | -1.8 | -2.3 | 0.008 | -2.5 | <0.001 | -10.4 | <0.001 |
| Ambp | -1.8 | 1.0 | 0.325 | 1.0 | 0.999 | -1.9 | <0.001 |
| C3 | -1.9 | -1.1 | 0.572 | -1.1 | 0.020 | -1.4 | 0.001 |
| Ldha | -2.2 | -1.4 | 0.138 | -1.6 | 0.030 | NP | NP |
| Mbl1 | -2.4 | 1.0 | 0.475 | -1.1 | 0.398 | -1.7 | <0.001 |
| Cp | -2.5 | 1.5 | 0.121 | 2.1 | <0.001 | -1.1 | 0.807 |
| Armet | -3.1 | NP | NP | NP | NP | NP | NP |
| Serpina3k | -3.3 | NP | NP | NP | NP | NP | NP |
| Mup1 | -4.2 | NP | NP | NP | NP | NP | NP |
| Hpx | -5.0 | -1.1 | 0.025 | -1.1 | 0.002 | NP | NP |
| Orm1 | -6.1 | -1.2 | 0.170 | -1.6 | 0.001 | -2.0 | <0.001 |
